# 6DOF knee kinematic alterations due to increased load levels

**DOI:** 10.3389/fbioe.2022.927459

**Published:** 2022-09-21

**Authors:** Tao Yang, Yaxiang Huang, Guoqing Zhong, Lingchuang Kong, Yuan Yan, Huahao Lai, Xiaolong Zeng, Wenhan Huang, Yu Zhang

**Affiliations:** ^1^ Department of Orthopaedics, Guangdong Provincial People’s Hospital, Guangdong Academy of Medical Sciences, Guangzhou, China; ^2^ Guangdong Key Lab of Orthopedic Technology and Implant Materials, Key Laboratory of Trauma & Tissue Repair of Tropical Area of PLA, Hospital of Orthopaedics, General Hospital of Southern Theater Command of PLA, Guangzhou, China; ^3^ Department of Orthopaedics, The First People’s Hospital of Jiujiang, Affiliated Jiujiang Hospital of Nanchang University, Jiujiang, China; ^4^ School of Medicine, South China University of Technology, Guangzhou, China

**Keywords:** load carriage, 6DOF kinematics, knee, gait, motion analysis

## Abstract

Whether load carriage leads to six-degrees-of-freedom (6DOF) knee kinematic alterations remains unclear. Exploring this mechanism may reveal meaningful knee kinematic information that can be used to improve load carriage conditions, the design of protective devices, and the knowledge of the effects of load carriage on knees. We recruited 44 subjects to explore kinematic alterations from an unloaded state to 60% bodyweight (BW) load carriage. A three-dimensional gait analysis system was used to collect the knee kinematic data. One-way repeated analysis of variance (ANOVA) was used to explore the effects of load levels on knee kinematics. The effects of increasing load levels on knee kinematics were smooth with decreased or increased trends. We found that knees significantly exhibited increased lateral tibial translation (up to 1.2 mm), knee flexion angle (up to 1.4°), internal tibial rotation (up to 1.3°), and tibial proximal translation (up to 1.0 mm) when they went from an unloaded state to 60%BW load carriage during the stance phase (*p* < 0.05). Significant small knee adduction/abduction angle and posterior tibial translation alterations (<1°/mm) were also identified (*p* < 0.05). Load carriage can cause significant 6DOF knee kinematic alterations. The results showed that knee kinematic environments are challenging during increased load. Our results contain kinematic information that could be helpful for knee-protection-related activities, such as target muscle training to reduce abnormal knee kinematics and knee brace design.

## 1 Introduction

Load carriage during walking is one of the most frequent human activities. The knee is reported to be the third-most frequent site of injury during load carriage exercises or training (Orr et al., 2015). It is reported that load carriage is one of the major causes of knee injuries, and it accounts for up to 15% of knee injuries in soldiers’ military training with loads up to about 50 kg (Knapik et al., 1992; Reynolds et al., 1999; Knapik, Reynolds, and Harman, 2004; Roy et al., 2012a; Roy et al., 2012b; Seay, 2015). There have been few investigations into knee diseases due to load carriage in daily activities. However, the relationship between knee injuries and load carriage among soldiers may still provide a meaningful reference for the relationship between load carriage and knee injuries. Lincoln et al. found that knee injuries accounted for about 48.8% of musculoskeletal disorders in soldiers (high load-demanding people, a total of 15,268 subjects), including meniscus injuries (about 24.2%), cruciate ligament injury (about 14.8%), collateral ligament injury (about 3.7%), and chondromalacia (about 6.0%) (Lincoln et al., 2002). Tennent et al. also documented that the rate of ACL injuries in the U.S. military was ten times higher than that of the average population. In addition, load carriage was reported to be further related to the development of knee osteoarthritis (Drew, Krammer, and Brown, 2021).

Scholars have suggested that load carriage can cause abnormal joint kinematics, which may affect the knee (Dames and Smith, 2016; Loverro, Hasselquist, and Lewis, 2019). Some of the human characteristics of knee joint kinematics during load carriage while walking has been identified by researchers. Attwells et al. (2006) found that knee flexion at heel strike increased in the stance phase under 40–50 kg of loading carriage. Chow et al. (2005) found that the knee flexion of adolescent girls at loading response increased with load level from 10–15% body weight (BW). Increased knee flexion in the stance phase has been theorized to be a protective strategy used to absorb great load forces to prevent knees from injury (Attwells et al., 2006; Majumdar, Pal, and Majumdar, 2010). Although these findings suggest that load carriage could cause abnormal kinematic alterations, there have been few investigations into the six-degrees-of-freedom (6DOF) knee kinematic alterations that occur during load carriage, including knee flexion/extension, external/internal rotation, adduction/abduction, posterior/anterior translation, proximal/distal translation, and medial/lateral translation (Zhang et al., 2015). Exploring the knee kinematic alterations during load carriage may provide a holistic view of the kinematic effects of load carriage on the knee joint.

However, whether load carriage leads to multiplanar knee kinematic alterations remains unclear. We hypothesized that load carriage causes abnormal 6DOF knee kinematic alterations. We explored 6DOF knee kinematic alterations under several increasing load carriage levels up to 60% BW. Our results may deepen the knowledge of the mechanisms that cause 6DOF kinematic alterations during load carriage and meaningful kinematic information for knee-protection-related activities, such as loading training, protective device design, and improving loading conditions.

## Methods

### Subjects

Healthy subjects between 18 and 30 years old were recruited for the study. They had the habit of engaging in moderate exercise (e.g., jogging) at least once a week for at least 30 min. The exclusion criteria were used to exclude people with the following: 1) a body mass index of greater than 30, 2) an injury or surgery in the hip, knee, or ankle, 3) a history of trauma in the lower limbs (e.g., fracture), 4) neuropathic diseases, 5) muscular diseases, 6) the inability to physically or mentally complete the procedure, 7) proneness to falling, or 8) any other condition that can influence the gait parameters of the lower limbs. Before study initiation and subject recruitment, the study protocol was approved by the Institutional Review Board of the Hospital of Orthopedics of the General Hospital of Southern Theater Command. A total of 44 healthy subjects (22 males and 22 females) were recruited for this study. The average participant age, height, and weight were 24.2 ± 3.1 years, 167.2 ± 8.7 cm, and 58.1 ± 9.7 kg, respectively. The average participant body mass index was 20.7 ± 2.1 kg/m^2^.

### Devices and experiment procedures

Knee joint kinematic data about the subjects were collected using a gait analysis system (Opti_Knee, Innomotion Inc, Shanghai, China) composed of a working station computer, a surgical navigation stereo infrared tracking device, a high-speed camera, a hand-held digitizing probe, two sets of markers, and a bi-directional treadmill (Zhang et al., 2015). The surgical navigation stereo infrared tracking device has a sampling frequency of 60 Hz and measurement accuracy of 0.3 mm root mean square (Elfring, de la Fuente, and Radermacher, 2010). The femur and tibia coordinate system was based on landmarks and previously reported. (Zhang et al., 2015). A high-speed camera (Basler aca640-90uc; Basler AG, Germany) was used to record and simultaneously identify gait cycles while collecting kinematic data. The collected kinematic data for all gait cycles were averaged using the custom software of the gait system (Opti_knee, v1.0.0). The collected data included knee flexion/extension angle (°), knee abduction/adduction angle (°), internal/external tibial rotation angle (°), tibial anterior/posterior translation (mm), tibial proximal/distal translation (mm), and tibial lateral/medial translation (mm).

**FIGURE 1:**
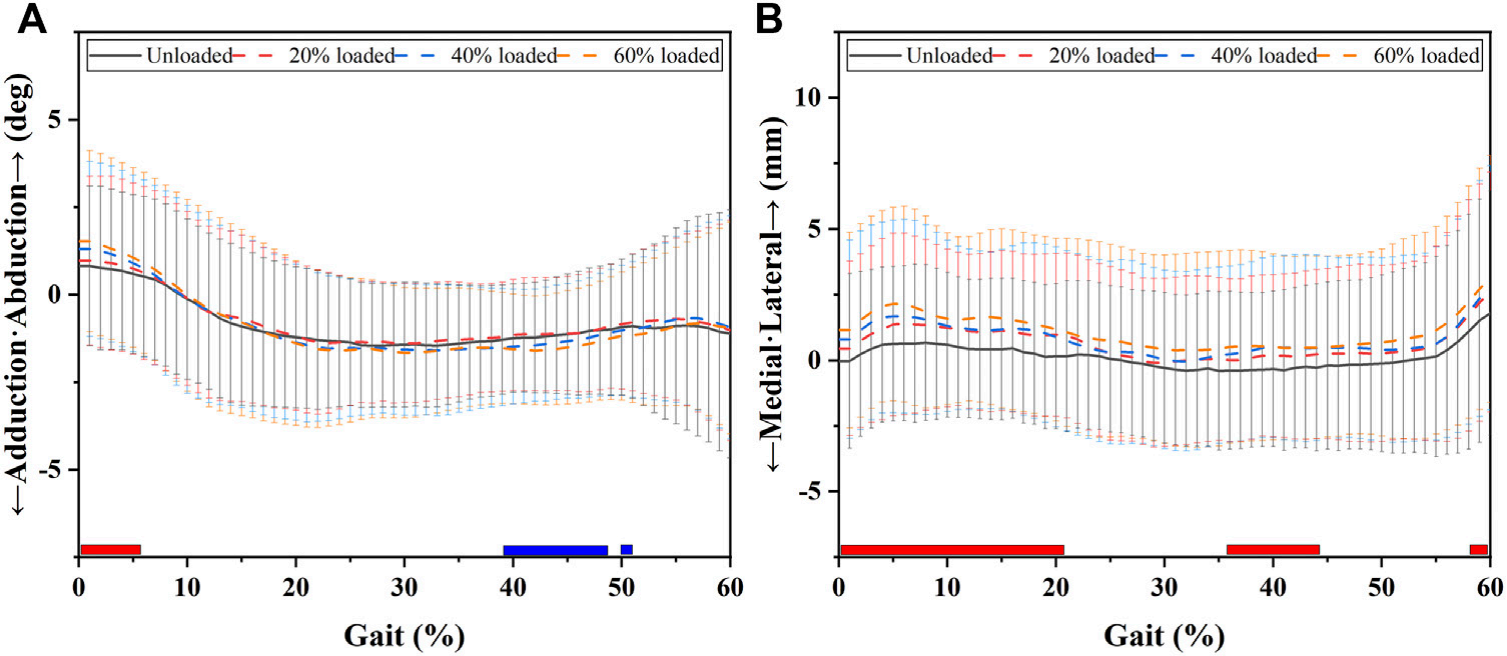
Coronal knee kinematic alterations during the stance phase with increased load levels. Chart **(A)** shows adduction (-)/abduction (+) alterations during the stance phase. Chart **(B)** shows medial (-)/lateral (+) tibial translation alterations during the stance phase. The blue bar shows when increased load levels significantly increased the adduction angle or medial tibial translation in the located phases. The red bars show the phases in which increased load levels significantly increased abduction angles or lateral tibial translation.

**FIGURE 2:**
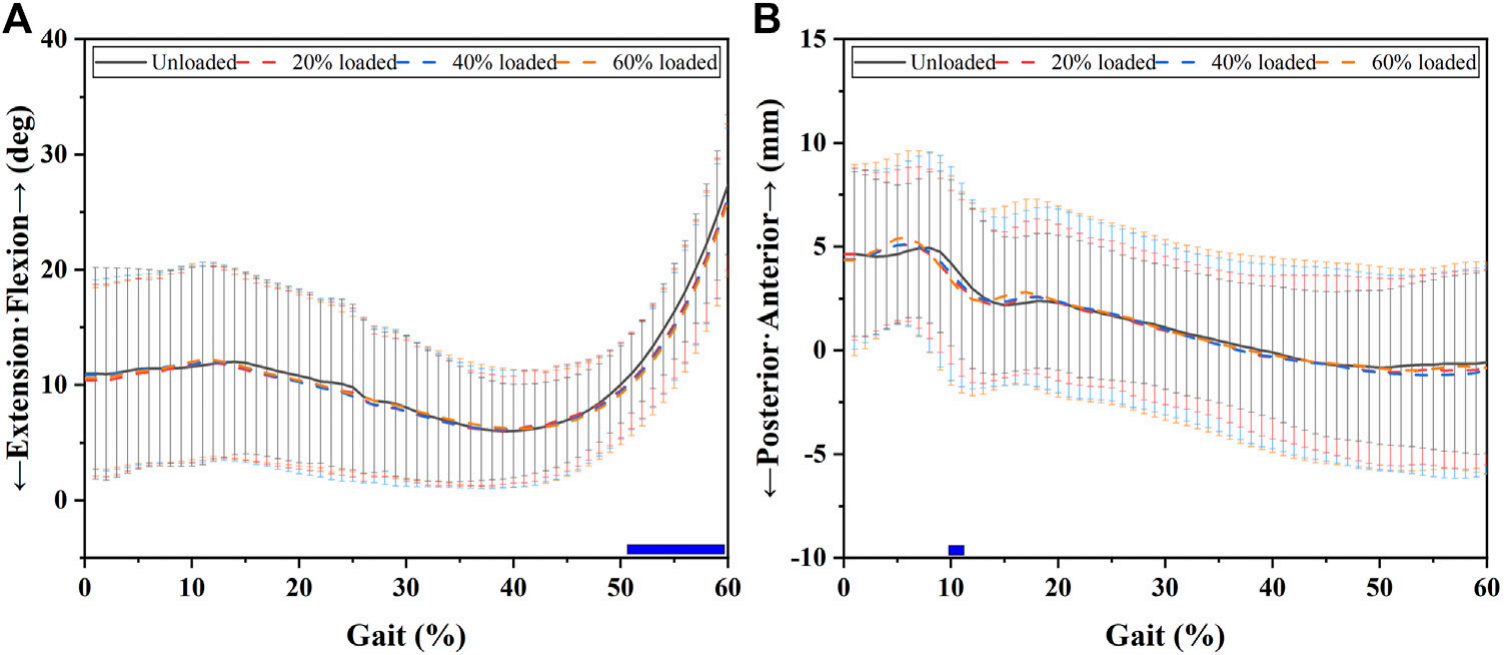
Sagittal knee kinematic alterations during the stance phase under increased load levels. Chart **(A)** shows the flexion (+)/extension (-) alterations that occurred during the stance phase. Chart **(B)** shows the anterior (+)/posterior (-) tibial translation alterations that occurred during the stance phase. The blue bars in the charts show the phases in which the increased load levels significantly increased the extension angle or posterior tibial translation.

Previous studies showed the level of load carriage that significantly changes kinematics was 20% or more of one’s BW (Attwells et al., 2006; Birrell and Haslam, 2009; Wang et al., 2013). Accordingly, the subjects would carry a weight vest with four loading levels while testing: unloaded and 20, 40, and 60% of the subject’s BW. This experiment lasted six days, and the subjects were not allowed to engage in load carriage tasks or sports besides daily activities for the duration of the experiment. The subjects only wore one level of weight vest each day. The experimental procedures were as follows: 1) the subjects with unloaded vests stood on the treadmill in a neutral position (Figure 1B); 2) two sets of four markers were fastened to the middle of the thigh and the middle of the shank; 3) a hand-held digitizing probe was used to identify seven bony markers (medial malleolus, lateral malleolus, medial epicondyle, lateral epicondyle, greater trochanter, lateral plateau, and medial plateau) to establish a personalized three-dimensional (3D) coordinate systems of the each participant’s tibia and femur; 4) subjects carried pre-selected loads and walked for 10 min to adapt to a barefoot walking speed of 3 km/h; 5) the gait system collected the knee kinematic data as the subjects walked on the bi-directional treadmill for 15 s; and 6) after full recovery for 1 day, the subjects were tested again with randomly-selected load levels until the experiments for all load levels were performed. Knee injuries often happen at the moment of ground contact (Hughes and Watkins, 2006; Swenson et al., 2013; Mitchell et al., 2016). The kinematic data collection for each test lasted for 15 s, and at least 15 gait cycles were collected and automatically identified and averaged by the custom software of the gait system. 1–60% gait phase was generally considered to be the stance phase of a gait cycle. Hence, 6DOF kinematics were analyzed during the stance phase (1–60% of the gait cycle, GC).

### Statistical analysis

**FIGURE 3:**
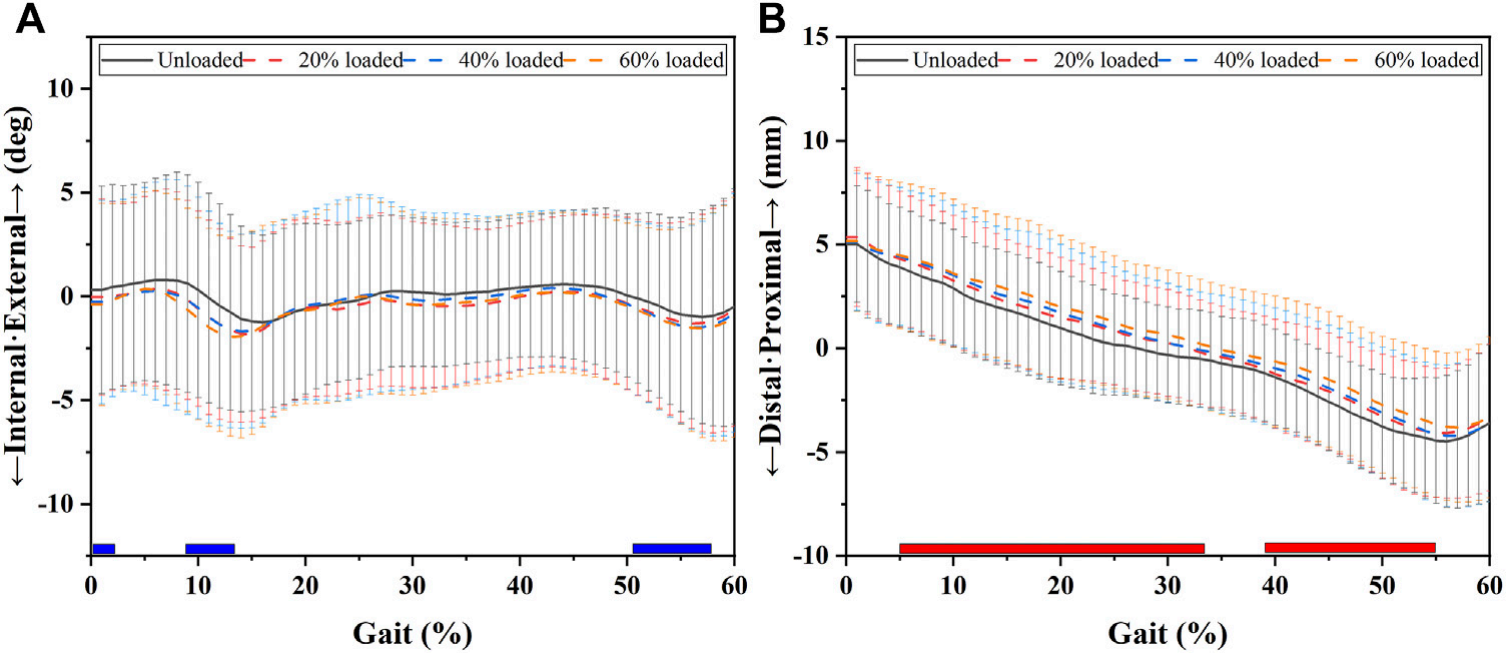
Transverse knee kinematic alterations during the stance phase with increased load levels. Chart **(A)** shows the internal (-)/external (+) tibial rotation alterations that occurred during the stance phase. Chart **(B)** shows the distal (-)/proximal (+) tibial translation alterations that occurred during the stance phase. The blue bars in Chart **(A)** show the phases in which increased load levels significantly increased internal tibial rotation angle or distal tibial translation. The red bars in Chart **(B)** show the phases in which increased load levels significantly increased the external tibial rotation angle or proximal tibial translation.

**FIGURE 4:**
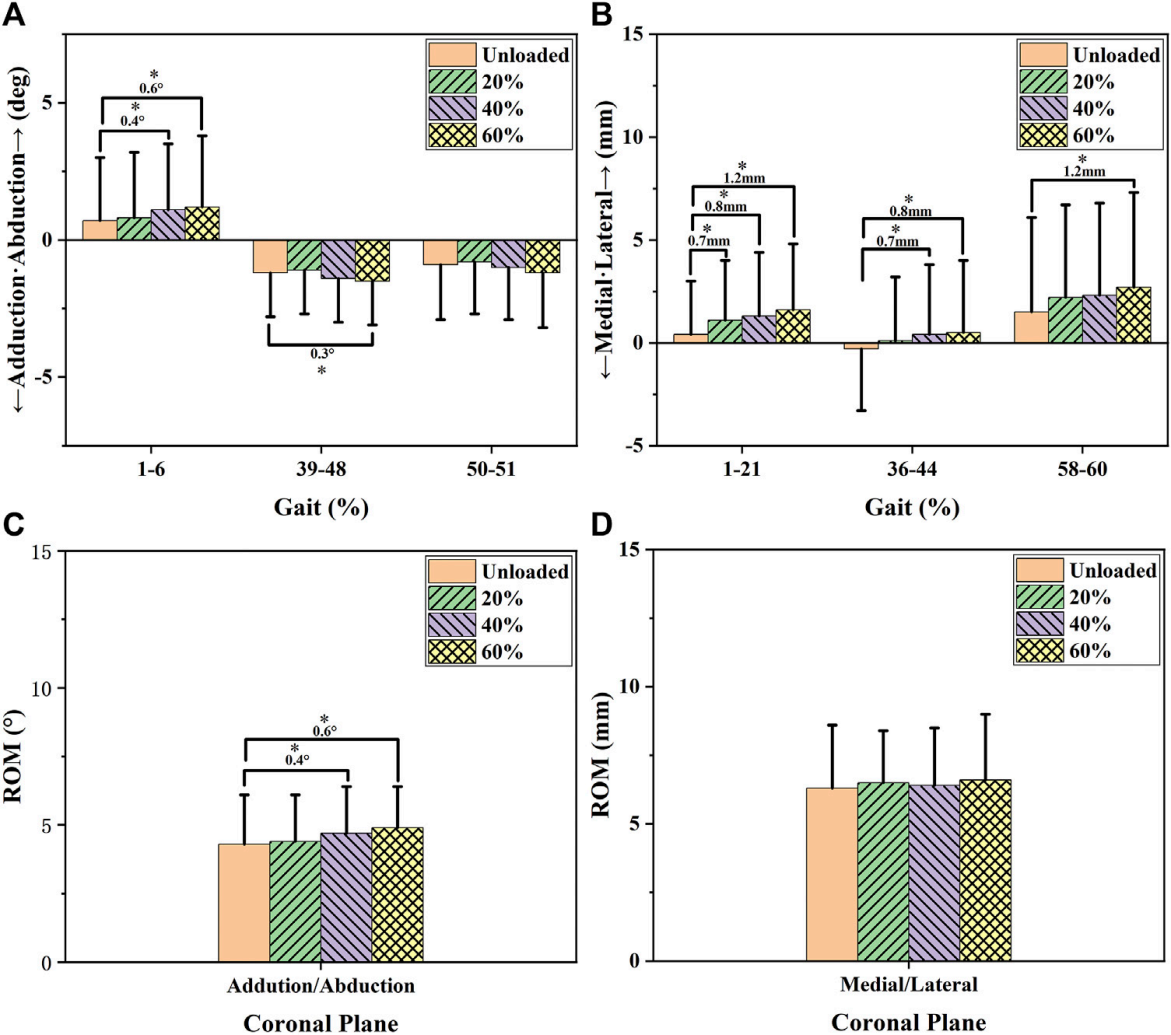
Coronal ROM and average knee kinematic alterations during the phases affected by increased load levels. Charts **(A** and **B)** show the average knee kinematics of the affected phases (i.e., the phases marked by colored bars in Figure 2) under increased load levels. Charts **(C** and **D)** show the ROM of coronal knee kinematics in increased load levels. The differences in knee kinematics between the unloaded level and load levels of 20, 40, and 60%BW were compared using a statistical significance level of 0.05. The number and * in the chart represent the differences in magnitude and significant differences between the groups (*p* < 0.05).

One-way repeated-measures analysis of variance (ANOVA), post-hoc analysis, and LSD (least significant difference) methods were performed on kinematic data. One-way repeated-measures ANOVA was first conducted to determine which parts of the stance phases involved in knee kinematics were significantly affected by load carriage (Figures 1 and 3). Then, the average knee kinematics of the affected phases and range of motion (ROM) of the stance phase during increased load levels were compared *via* one-way repeated-measures ANOVA and LSD methods. The kinematic comparisons between unloaded and different load levels are shown in Figures 4 and 6.

The first five males and five females were recruited for sample size calculation *via* PASS version 15.0 (NCSS, LLC. Kaysville, Utah, United States). The ROM of the adduction angle was selected to calculate the sample size due to the effect of adduction on a knee injury and osteoarthritis development (Zhang and Wang, 2001; Sun et al., 2017). The mean standard deviations for the ROM of the adduction angle under increasing load levels were 4.0 ± 1.5°, 4.1 ± 1.2°, 4.9 ± 1.6°, and 5.0 ± 1.3° from unloaded to 60% BW conditions among the ten subjects. The power (1-β) and alpha were set to 80% and 0.05, respectively. Accordingly, a sample size of 17 subjects could achieve 82.31% power to detect differences between various load levels with a significance level of 0.05 using the one-way repeated-measures module in PASS version 15.0 (NCSS LLC. Kaysville, Utah, United States). Hence, we finally recruited 44 subjects, which was enough to meet the sample size requirement of at least 17 subjects.

## Results

The effects of increased load levels on 6DOF knee kinematics are exhibited in Figures 1–3. The average knee kinematics values of the affected phases and ROM of the stance phase during increased load levels are exhibited in Figures 4 and 6 and Table 1.

**TABLE 1:** Temporospatial parameters and kinematic comparison of the affected gait phases during load carriage.

	Unloaded	20% BW	40% BW	60% BW	F Value	*p*-value
**Adduction/abduction (degree)**						
1–6%GC	0.7 ± 2.3	0.8 ± 2.4	1.1 ± 2.5*	1.2 ± 2.6*	8.811	<0.001
39–48%GC	−1.2 ± 1.6	−1.1 ± 1.6	1.4 ± 1.6	−1.5 ± 1.6*	6.317	0.006
50–51%GC	−0.9 ± 2.0	−0.8 ± 1.9	−1.0 ± 1.9	−1.2 ± 1.9	3.533	0.046
ROM	4.3 ± 1.8	4.4 ± 1.7	4.7 ± 1.7*	4.9 ± 1.5*	6.865	0.001
**Tibial lateral/medial translation (mm)**						
1–12%GC	0.4 ± 2.6	1.1 ± 2.9*	1.3 ± 3.1*	1.6 ± 3.2*	11.480	<0.001
26–44%GC	−0.3 ± 3.0	0.1 ± 3.1	0.4 ± 3.4*	0.5 ± 3.5*	4.017	0.030
58–60%GC	1.5 ± 4.6	2.2 ± 4.5	2.3 ± 4.5*	2.7 ± 4.6*	4.259	0.031
ROM	6.3 ± 2.3	6.5 ± 1.9	6.4 ± 2.1	6.6 ± 2.4*	0.476	0.615
**Flexion/extension (degree)**						
51–60%GC	18.0 ± 4.2	17.0 ± 4.9*	16.8 ± 4.8*	16.6 ± 5.3*	7.820	0.001
ROM	29.4 ± 6.2	28.5 ± 6.3	28.2 ± 6.6	28.1 ± 7.1	1.771	0.168
**Tibial anterior/posterior translation (mm)**						
10–11%GC	3.9 ± 4.0	**3.3** ± 4.2*	3.4 ± 5.0	3.1 ± 4.9*	3.251	0.033
ROM	9.3 ± 4.2	9.5 ± 3.9	9.9 ± 4.2	9.9 ± 4.0	1.880	0.155
**Tibial internal/external rotation (degree)**						
1–2%GC	0.4 ± 4.9	0 ± 4.6	−0.2 ± 4.8*	−0.3 ± 4.7*	3.459	0.027
8–13%GC	0.1 ± 5.0	**−0.8** ± 4.6*	−0.8 ± 5.1*	−1.2 ± 4.7*	6.884	0.001
51–57%GC	−0.6 ± 4.5	**−1.0** ± 4.6*	−1.2 ± 4.6*	−1.2 ± 4.6*	4.587	0.011
ROM	9.0 ± 8.5	8.5 ± 3.2	8.8 ± 3.3	8.8 ± 3.5	1.230	0.301
**Tibial distal/proximal translation (mm)**						
5–33%GC	1.3 ± 2.5	**1.8** ± 2.8*	2.0 ± 3.0*	2.2 ± 3.2*	8.083	0.001
39–55%GC	−3.0 ± 2.4	−2.6 ± 2.8	−2.4 ± 3.0*	−2.0 ± 3.2*	7.847	0.001
ROM	10.4 ± 3.0	10.4 ± 3.2	10.3 ± 3.3	10.0 ± 3.1	1.447	0.238
**Cadence (steps/min)**	108.5 ± 1.9	105.7 ± 1.8	106.1 ± 1.4*	**105.4** ± 1.5*	3.217	**0.040**
**Step length (cm)**	41.1 ± 6.2	43.0 ± 5.5	41.9 ± 7.3	42.7 ± 5.7	1.468	0.233

* Significant difference (<0.05) compared to unloaded walking by LSD methods.

Statistical methods: One-way repeated ANOVA

**FIGURE 5:**
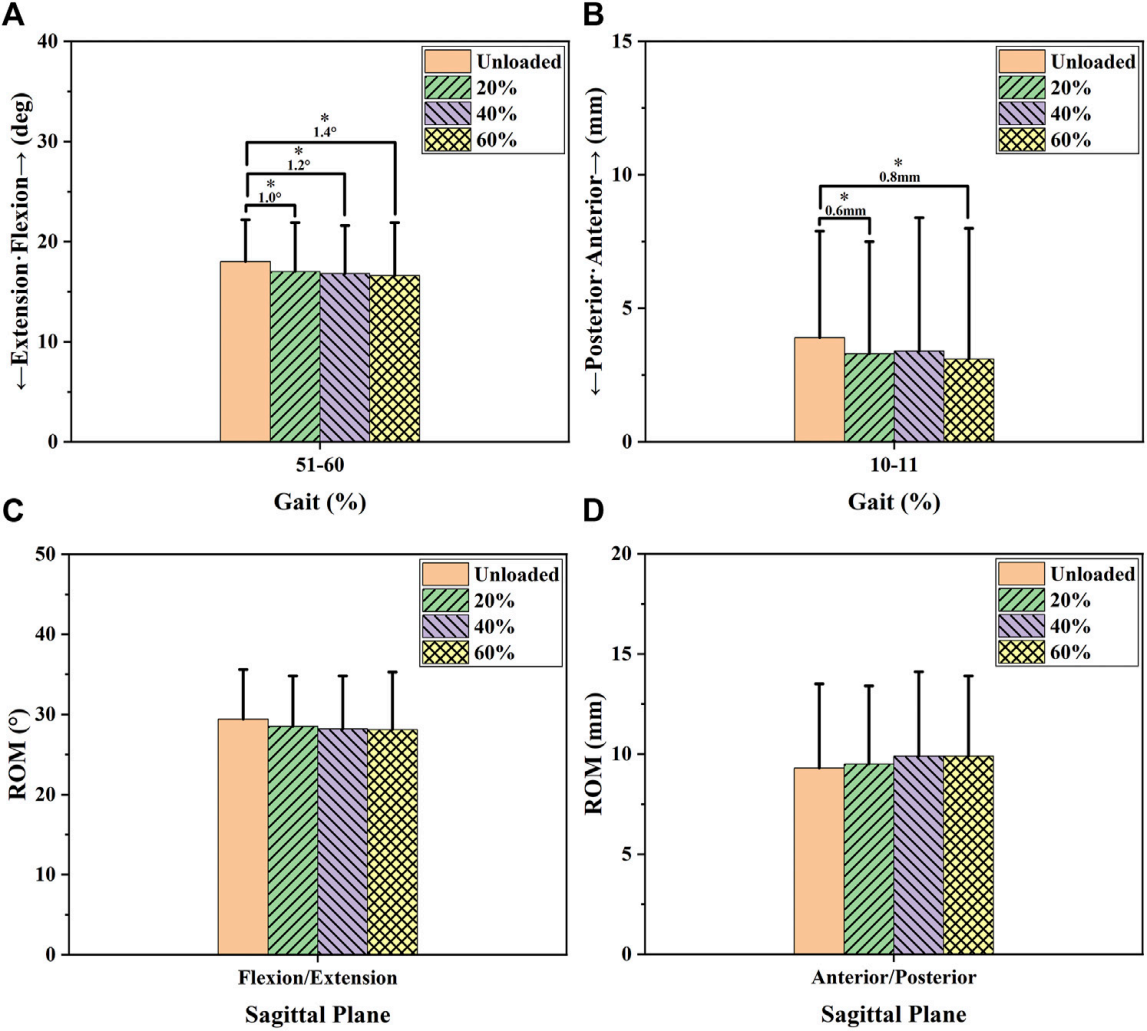
Sagittal ROM and average knee kinematic alterations during the phases affected by increased load levels. Charts **(A** and **B)** show the average knee kinematics of the affected phases (i.e., the phases marked by colored bars in Figure 2) under increased load levels. Charts **(C** and **D)** show the ROM of sagittal knee kinematics under increased load levels. The differences in the knee kinematics between the unloaded state and load levels of 20, 40, and 60%BW were compared using a statistical significance level of 0.05. The number and * in the chart represent the differences in magnitude and significant differences between the groups (*p* < 0.05).

**FIGURE 6:**
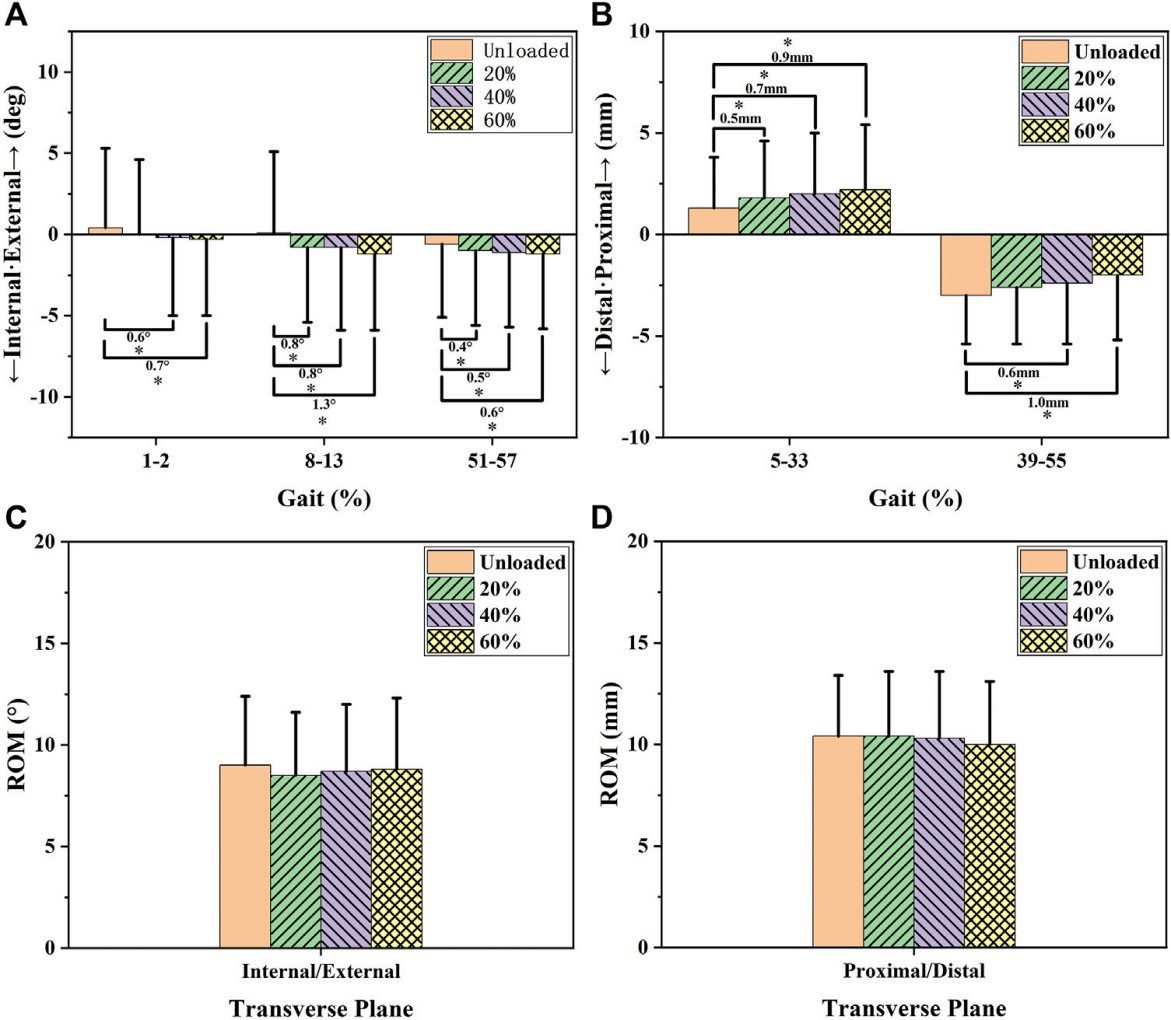
Transverse ROM and average knee kinematic alterations during the phases affected by increased load levels. Charts **(A** and **B)** show the average knee kinematics of the affected phases (i.e., the phases marked by colored bars in Figure 2) under increased load levels. Charts **(C** and **D)** show the ROM of the transverse knee kinematics under increased load levels. The differences in the knee kinematics between the unloaded state and load levels of 20, 40, and 60%BW were compared using a statistical significance level of 0.05. The number and * in the chart represent differences in magnitude and significant differences between the groups (*p* < 0.05).

All the kinematic data comparisons in the table are drawn in Figures 4–6.

The bold font and * was both to highlight the kinematics was significantly (*p* < 0.05) from those of unloaded status.

### Coronal knee kinematics under increasing load conditions

Figures 1 and 4 and Table 1 show the effects of increased load levels on coronal knee kinematics consisting of adduction/abduction angle changes and medial/lateral tibial translation. Increased load levels led to increased abduction angles during 1–6% of the GC, whereas they led to increased adduction angles in 39–48% and 50–51% of the GC (*p* < 0.05, see Figure 1A). Increased load levels increased the average knee abduction angle during 1–6% of the GC with 0.4–0.6° under the level of statistical significance (*p* < 0.05, Figure 4A). Increased load levels increased the average knee adduction angle during 39–48% of the GC with 0.3° under the level of statistical significance (*p* < 0.05, Figure 4A). The increased load level also increased the ROM of the adduction angle during the stance phase by 0.4–0.6° (*p* < 0.05, Figure 4C). In conclusion, the effects of increased load levels on adduction/abduction angle were significant but small (<1°).

Increased load levels led to increased lateral tibial translation in 1–21%, 36–44%, and 58–60% of the GC (*p* < 0.05, Figure 1B). Increased load levels increased the average lateral tibial translation during 1–21% of the GC with 0.7–1.2 mm under the level of statistical significance (*p* < 0.05, Figure 4B). Increased load levels increased the average lateral tibial translation during 36–44% of the GC with 0.7–0.8 mm under the level of statistical significance (*p* < 0.05, Figure 4B). Increased load levels increased the average of lateral tibial translation during 58–60% of the GC with 1.2 mm under the level of statistical significance (*p* < 0.05, Figure 4B). Overall, the effects of increased load levels on lateral tibial translation were significant with up to 1.2 mm increment.

### Sagittal knee kinematics under increasing load conditions

Figures 2 and 5 and Table 1 show the effects of increased load level on sagittal knee kinematics consisting of flexion/extension angle and anterior/posterior tibial translation. Increased load levels led to increased extension angles in 51–60% of the GC (*p* < 0.05, Figure 2A). Increased load levels increased the average knee extension angle during 51–60% of the GC with 1.0–1.4° under the level of statistical significance (*p* < 0.05, Figure 5A). In conclusion, the effects of increased load levels on extension angle were significant with up to 1.4° increment.

Increased load levels led to increased posterior tibial translation in 10–11% of the GC (*p* < 0.05, Figure 2B). Increased load levels increased the average of posterior tibial translation during 10–11% of the GC with 0.6–0.8 mm under the level of statistical significance (*p* < 0.05, Figure 5B). Overall, the effects of increased load levels on posterior tibial translation were significant but small (<1 mm).

### Transverse knee kinematics under increasing load conditions

Figures 3 and 6 and Table 1 show the effects of increased load levels on transverse knee kinematics consisting of internal/external tibial rotation angle and distal/proximal tibial translation. Increased load levels led to increased tibial rotation angles in 1–2%, 8–13%, and 51–57% of the GC (*p* < 0.05, Figure 3A). Increased load levels increased the average internal tibial rotation angle during 1–2% of the GC with 0.6–0.7° under the level of statistical significance (*p* < 0.05, Figure 6A). Increased load levels increased the average internal tibial rotation angle during 8–13% of the GC with 0.8–1.3° under the level of statistical significance (*p* < 0.05, Figure 6A). Increasing the load level increased the average internal tibial rotation angle during 51–57% of GC with 0.4–0.6° under the level of statistical significance (*p* < 0.05, Figure 6A). In conclusion, the effects of increased load levels on internal tibial rotation angle were significant with up to 1.3° increment.

Increased load levels led to increased proximal tibial translation in 5–33% and 39–56% of the GC (*p* < 0.05, Figure 3B). Increased load levels increased the average of proximal tibial translation during 5–33% of the GC with 0.5–0.9 mm under the level of statistical significance (*p* < 0.05, Figure 6B). Increased load levels increased the average proximal tibial translation during 39–55% of the GC with 0.6–1.0 mm under the level of statistical significance (*p* < 0.05, Figure 6B). Overall, the effects of increased load levels on proximal tibial translation were significant with up to 1.0 mm increment.

## Discussion

Whether load carriage alters 6DOF knee kinematics remains unclear. We attempted to fill this gap in this study. Our results confirmed our hypothesis and showed that load carriage smoothly causes 6DOF knee kinematic alterations with increased load levels. We found that increased load levels increased lateral tibial translation (up to 1.2 mm), knee flexion angle (up to 1.4°), internal tibial rotation (up to 1.3°), and tibial proximal translation (up to 1.0 mm) from unloaded to 60% BW during stance phases (*p* < 0.05, Figures 1 and 6). However, significant minor alterations (<1°/mm) of adduction angle and posterior tibial translation were also found between the unloaded state and a load of 60% of the participant’s BW (*p* < 0.05). This study showed the knee kinematics alterations that occurred due to load carriage increases. It may provide a reference for people who design activities for improving knee conditions during load carriage, such as special training programs before load carriage tasks, and knee braces or protective devices related to load-bearing/carriage.

In the coronal plane, increased load levels increased lateral tibial translation (0.7–1.2mm, Figure 1B; Figure 4B). No research has been performed to explore the effects of lateral tibial translation during load carriage. However, Li et al. showed the roles of medial/lateral translation on knee joint movements (Li et al., 2007). They suggested that the increased medial tibial translation could shift the joint contact in the medial compartment toward the medial tibial spine of the knee joint and result in cartilage degeneration in patients with ACL deficiencies (Li et al., 2007). Similarly, increased lateral tibial translation during load carriage can shift the joint contact in the lateral compartment toward the lateral tibial spine of the knee joint and result in cartilage degeneration during long-term load carriage tasks. Significant adduction and abduction alterations were also found under increased load levels (Figures 1A, 4A and 4C). Both knee adduction and abduction angles were reported to play an important role in knee disease development in previous literature. Crema et al. (2012) found that abduction or adduction malalignment (<178° or >182°) can be related to meniscus extrusion and, thus, increased rates of meniscus tearing. Mehl et al. (2018) declared that knee abduction and low flexion angles (5–20°) increased the risk of ACL injury. Under this condition, the *in-situ* ACL forces significantly increased, and the lateral femoral condyle impinged ACL movement (Mehl et al., 2018). Furthermore, Shin et al. found that internal tibial rotation and knee abduction can further increase ACL strain and the risk of ACL injury (Shin, Chaudhari, and Andriacchi, 2011). However, the adductive and abductive angular changes observed in the present study were small (<1°) but significant. Perhaps, small adductive or abductive alterations take a long time to significantly affect knee health.

In the sagittal plane, increased load levels led to increased knee extension angles (1.0–1.4°) during the terminal stance phase (51–60% GC, Figures 2A and 5A). Increased knee extension angles may improve knee stability during the terminal stance phase due to the screw-home mechanism (Moglo and Shirazi-Adl, 2005). However, other researchers reported different results. Talarico et al. reported that flexion at heel strike (initial contact) increased with increasing load levels and declared that high knee flexion angles at heel strike might increase the stability of the knee and allow the knee to absorb high loads (Talarico et al., 2018). The two kinds of knee flexion/extension response may both protect the knee joint. Increased posterior tibial translation (0.6–0.8 mm) was found in 10–11% of the GC (*p* < 0.05, Figures 2B and 5B). Having an abnormal anteroposterior tibial position was considered to be a risk factor for cartilage degeneration or the progression of osteoarthritis in knee joints (Zaid et al., 2015; Kiapour, Fleming, and Murray, 2017; Ikuta et al., 2020; Li et al., 2020). For example, Ikuta et al. found that posterior tibial translation increased during knee extension–flexion cycles in the sitting and squatting positions as knee osteoarthritis progressed (Ikuta et al., 2020). Li et al. found that increased anterior tibial translation 6 months after surgery was correlated with cartilage degeneration in the medial tibia plateau at the 1-year and 2-year follow-ups with patients with unloaded ACL-reconstructed knees between full extension and 30° of flexion *via* a magnetic resonance imaging (MRI) device (Li et al., 2020). However, the increased posterior tibial translation (0.6–0.8 mm) alterations that occurred under increased load levels were small (<1 mm). Similarly, like the adduction/abduction alterations, increased posterior tibial translation may take a long time to greatly affect knee health.

In the transverse plane, increased load levels led to increased tibial rotation angles (up to 1.3°) in 1–2%, 8–13%, and 51–57% of the GC (*p* < 0.05, Figures 3A and 6A). Increased tibial rotation was reported to be associated with knee-related injuries, such as overuse injuries and tendinopathy (Shin, Chaudhari, and Andriacchi, 2011; Mousavi et al., 2019; De Bleecker et al., 2020). In a meta-analysis, consistent with the results of our study, Bleecker et al. found that increased internal knee rotation at initial contact (1–2% of the GC) was significantly positively correlated with knee overuse injuries compared to healthy controls (De Bleecker et al., 2020). Shin et al. (2011) found that internal tibial rotation and knee abduction can increase ACL strain and the risk of ACL injury. Therefore, increased internal tibial translation due to load carriage could potentially make the knee joint vulnerable. Increased proximal tibial translation (up to 1.0 mm) was found under increased load levels (Figures 3B and 6B). The increased proximal tibial translation was the description of the narrowed knee joint space. The narrowed knee joint space could be a result of the increased load force on the knee joint during load carriage (Simpson, Munro, and Steele, 2012; Dames and Smith, 2016). The increased force could squeeze the knee cartilage or meniscus and increase proximal tibial translation. Consistent with our study, Sutter et al. used MRI to detect the thickness of knee cartilage after single-leg hops and found that the knee cartilage was compressed by 2–6% by the extra force of single-leg hops (Sutter et al., 2019). Compressed cartilage can be injured under sustained load carriage conditions (Forster and Fisher, 1999).

However, the present study has some limitations. We did not explore hip and ankle kinematic parameters, which may also play important roles in load carriage (Dames and Smith, 2016; Liew, Morris, and Netto, 2016; Tian et al., 2018). The kinematic alterations during increased load levels were relatively small. Considering the measurement accuracy of the gait system (0.3 mm), significant average translations of 0.5 mm or 0.5° can be considered important changes. As discussed previously, the results (Figure 6B) showed that proximal tibial translation increases as the load level is increased. This may be a result of a high joint load transmitting through the knee joint (Simpson, Munro, and Steele, 2012; Dames and Smith, 2016), resulting in the compression of joint cartilage and the meniscus. Nevertheless, we did not use a force plate to record the ground reaction force and MRI to directly observe joint cartilage deformation (Sutter et al., 2019). The effects of the load carriage on ground reaction force and joint cartilage deformation should be further explored.

## Conclusion

We found that increasing load levels led to 6DOF knee kinematic alterations. These alterations showed that the knee joints may be in an easy-to-injure state rather than a protective one (see Figures 1 and 6). The knee joints exhibited increased lateral tibial translation (up to 1.2 mm), knee flexion angle (up to 1.4°), internal tibial rotation (up to 1.3°), and tibial proximal translation (up to 1.0 mm) with increased load levels. Small but significant amounts of adduction/abduction and posterior tibial translation (<1°/mm) were also found under increased load levels. The findings could enhance our understanding of the 6DOF knee kinematic alteration mechanism during load carriage. This could provide meaningful information for designing protective devices, improving loading conditions, and loading training. For example, the designers of protective knee devices can strengthen the lateral support when designing knee braces for load carriage tasks due to the increased lateral translation (up to 1.2 mm) during the stance phase of the GC. Clinicians and trainers could make some suggestions (e.g., improving the medial/lateral muscle strength) for people who often undertake load carriage tasks.

## Data Availability

The data are available from the corresponding authors upon a reasonable request.

## References

[B1] Attwells R. L. Birrell S. A. Hooper R. H. Mansfield N. J. (2006). 'Influence of carrying heavy loads on soldiers' posture, movements and gait. Ergonomics 49, 1527–1537. 10.1080/00140130600757237 17050392

[B2] Birrell S. A. Haslam R. A. (2009). The effect of military load carriage on 3-D lower limb kinematics and spatiotemporal parameters. Ergonomics 52, 1298–1304. 10.1080/00140130903003115 19787507

[B3] Crema M. D. Roemer F. W. Felson D. T. Englund M. Wang K. Jarraya M. (2012). 'Factors associated with meniscal extrusion in knees with or at risk for osteoarthritis: The multicent er osteoarthritis study. Radiology 264, 494–503. 10.1148/radiol.12110986 22653191 PMC3401352

[B4] Dames K. D. Smith J. D. (2016). 'Effects of load carriage and footwear on lower extremity kinetics and kinematics during overground walking. Gait Posture 50, 207–211. 10.1016/j.gaitpost.2016.09.012 27649512

[B5] De Bleecker C. Vermeulen S. De Blaiser C. Willems T. De Ridder R. Roosen P. (2020). Relationship between jump-landing kinematics and lower extremity overuse injuries in physically active populations: A systematic review and meta-analysis. Sports Med. 50, 1515–1532. 10.1007/s40279-020-01296-7 32514700

[B6] Drew M. D. Krammer S. M. Brown T. N. (2021). 'Effects of prolonged walking with body borne load on knee adduction biomechanics. Gait Posture 84, 192–197. 10.1016/j.gaitpost.2020.12.004 33360641 PMC7902390

[B7] Elfring R. de la Fuente M. Radermacher K. (2010). 'Assessment of optical localizer accuracy for computer aided surgery systems. Comput. Aided Surg. 15, 1–12. 10.3109/10929081003647239 20233129

[B8] Forster H. Fisher J. (1999). 'The influence of continuous sliding and subsequent surface wear on the friction of articular cartilage. Proc. Inst. Mech. Eng. H. 213, 329–345. 10.1243/0954411991535167 10466364

[B9] Hughes G. Watkins J. (2006). 'A risk-factor model for anterior cruciate ligament injury. Sports Med. 36, 411–428. 10.2165/00007256-200636050-00004 16646629

[B10] Ikuta F. Yoneta K. Miyaji T. Kidera K. Yonekura A. Osaki M. (2020). 'Knee kinematics of severe medial knee osteoarthritis showed tibial posterior translation and external rotation: A cross-sectional study. Aging Clin. Exp. Res. 32, 1767–1775. 10.1007/s40520-019-01361-w 31598915

[B11] Kiapour A. M. Fleming B. C. Murray M. M. (2017). 'Structural and anatomic restoration of the anterior cruciate ligament is associated with less cartilage damage 1 Year after surgery: Healing ligament properties affect cartilage damage. Orthop. J. Sports Med. 5, 232596711772388. 10.1177/2325967117723886 PMC5576541 28875154

[B12] Knapik J. J. Reynolds K. L. Harman E. (2004). 'Soldier load carriage: Historical, physiological, biomechanical, and medical aspects. Mil. Med. 169, 45–56. 10.7205/milmed.169.1.45 14964502

[B13] Knapik J. Reynolds K. Staab J. Vogel J. A. Jones B. (1992). 'Injuries associated with strenuous road marching. Mil. Med. 157, 64–67. 10.1093/milmed/157.2.64 1603388

[B14] Li A. K. Ochoa J. K. Pedoia V. Amano K. Souza R. B. Li X. (2020). 'Altered tibiofemoral position following ACL reconstruction is associated with cartilage matrix changes: A voxel-based relaxometry analysis. J. Orthop. Res. 38, 2454–2463. 10.1002/jor.24708 32369216

[B15] Li G. Papannagari R. DeFrate L. E. Yoo J. D. Park S. E. Gill T. J. (2007). 'The effects of ACL deficiency on mediolateral translation and varus-valgus rotation. Acta Orthop. 78, 355–360. 10.1080/17453670710013924 17611849

[B16] Liew B. X. Morris S. Netto K. (2016). 'Joint power and kinematics coordination in load carriage running: Implications for performance and injury. Gait Posture 47, 74–79. 10.1016/j.gaitpost.2016.04.014 27264407

[B17] Lincoln A. E. Smith G. S. Amoroso P. J. Bell N. S. (2002). The natural history and risk factors of musculoskeletal conditions resulting in disability among US Army personnel. Work 18, 99–113. 12441574 PMC2151132

[B18] Loverro K. L. Hasselquist L. Lewis C. L. (2019). Females and males use different hip and knee mechanics in response to symmetric military-relevant loa ds. J. Biomech. 95, 109280. 10.1016/j.jbiomech.2019.07.024 31405526

[B19] Majumdar D. Pal M. S. Majumdar D. (2010). 'Effects of military load carriage on kinematics of gait. Ergonomics 53, 782–791. 10.1080/00140131003672015 20496244

[B20] Mehl J. Diermeier T. Herbst E. Imhoff A. B. Stoffels T. Zantop T. (2018). Evidence-based concepts for prevention of knee and ACL injuries. 2017 guidelines of the ligament comm ittee of the German Knee Society (DKG). Arch. Orthop. Trauma Surg. 138, 51–61. 10.1007/s00402-017-2809-5 28983841

[B21] Mitchell J. Graham W. Best T. M. Collins C. Currie D. W. Comstock R. D. (2016). 'Epidemiology of meniscal injuries in US high school athletes between 2007 and 2013. Knee Surg. Sports Traumatol. Arthrosc. 24, 715–722. 10.1007/s00167-015-3814-2 26506845 PMC5189670

[B22] Moglo K. E. Shirazi-Adl A. (2005). 'Cruciate coupling and screw-home mechanism in passive knee joint during extension--flexion. J. Biomech. 38, 1075–1083. 10.1016/j.jbiomech.2004.05.033 15797589

[B23] Mousavi S. H. Hijmans J. M. Rajabi R. Diercks R. Zwerver J. van der Worp H. (2019). Kinematic risk factors for lower limb tendinopathy in distance runners: A systematic review and meta-analysis. Gait Posture 69, 13–24. 10.1016/j.gaitpost.2019.01.011 30658311

[B24] Orr R. M. Johnston V. Coyle J. Pope R. (2015). 'Reported load carriage injuries of the Australian army soldier. J. Occup. Rehabil. 25, 316–322. 10.1007/s10926-014-9540-7 25178432

[B25] Reynolds K. L. White J. S. Knapik J. J. Witt C. E. Amoroso P. J. (1999). 'Injuries and risk factors in a 100-mile (161-km) infantry road march. Prev. Med. 28, 167–173. 10.1006/pmed.1998.0396 10048108

[B26] Roy T. C. Knapik J. J. Ritland B. M. Murphy N. Sharp M. A. (2012a). 'Risk factors for musculoskeletal injuries for soldiers deployed to Afghanistan. Aviat. Space Environ. Med. 83, 1060–1066. 10.3357/asem.3341.2012 23156094

[B27] Roy T. C. Ritland B. M. Knapik J. J. Sharp M. A. (2012b). 'Lifting tasks are associated with injuries during the early portion of a deployment to Afghanistan. Mil. Med. 177, 716–722. 10.7205/milmed-d-11-00402 22730849

[B28] Seay J. F. (2015). 'Biomechanics of load carriage--historical perspectives and recent insights. J. Strength Cond. Res. 29 (11), S129–S133. 10.1519/jsc.0000000000001031 26506175

[B29] Shin C. S. Chaudhari A. M. Andriacchi T. P. (2011). 'Valgus plus internal rotation moments increase anterior cruciate ligament strain more than either alo ne. Med. Sci. Sports Exerc. 43, 1484–1491. 10.1249/mss.0b013e31820f8395 21266934

[B30] Simpson K. M. Munro B. J. Steele J. R. (2012). 'Effects of prolonged load carriage on ground reaction forces, lower limb kinematics and spatio-temporal parameters in female recreational hikers. Ergonomics 55, 316–326. 10.1080/00140139.2011.642004 22409169

[B31] Sun H. Zhou L. Li F. Duan J. (2017). 'Comparison between closing-wedge and opening-wedge high tibial osteotomy in patients with medial knee osteoarthritis: A systematic review and meta-analysis. J. Knee Surg. 30, 158–165. 10.1055/s-0036-1584189 27218480

[B32] Sutter E. G. Liu B. Utturkar G. M. Widmyer M. R. Spritzer C. E. Cutcliffe H. C. (2019). 'Effects of anterior cruciate ligament deficiency on tibiofemoral cartilage thickness and strains in response to hopping. Am. J. Sports Med. 47, 96–103. 10.1177/0363546518802225 30365903 PMC6559720

[B33] Swenson D. M. Collins C. L. Best T. M. Flanigan D. C. Fields S. K. Comstock R. D. (2013). Epidemiology of knee injuries among U.S high school athletes, 2005/2006-2010/2011. Med. Sci. Sports Exerc. 45, 462–469. 10.1249/MSS.0b013e318277acca 23059869 PMC3768257

[B34] Talarico M. K. Haynes C. A. Douglas J. S. Collazo J. (2018). Spatiotemporal and kinematic changes in gait while carrying an energy harvesting assault pack system. J. Biomech. 74, 143–149. 10.1016/j.jbiomech.2018.04.035 29752054

[B35] Tian M. Park H. Li J. Koo H. Xu Q. (2018). 'Effects of load carriage and work boots on lower limb kinematics of industrial workers. Int. J. Occup. Saf. Ergon. 24, 582–591. 10.1080/10803548.2017.1334336 28693378

[B36] Wang H. Frame J. Ozimek E. Leib D. Dugan E. L. (2013). 'The effects of load carriage and muscle fatigue on lower-extremity joint mechanics. Res. Q. Exerc. Sport 84, 305–312. 10.1080/02701367.2013.814097 24261009

[B37] Zaid M. Lansdown D. Su F. Pedoia V. Tufts L. Rizzo S. (2015). 'Abnormal tibial position is correlated to early degenerative changes one year following ACL reconstruction. J. Orthop. Res. 33, 1079–1086. 10.1002/jor.22867 25721417 PMC7238841

[B38] Zhang L. Q. Wang G. (2001). 'Dynamic and static control of the human knee joint in abduction-adduction. J. Biomech. 34, 1107–1115. 10.1016/s0021-9290(01)00080-x 11506781

[B39] Zhang Y. Yao Z. Wang S. Huang W. Ma L. Huang H. (2015). 'Motion analysis of Chinese normal knees during gait based on a novel portable system. Gait Posture 41, 763–768. 10.1016/j.gaitpost.2015.01.020 25743776

